# Safety and feasibility of outpatient parenteral antimicrobial therapy for patients with spinal infection

**DOI:** 10.1038/s41598-023-33502-7

**Published:** 2023-04-26

**Authors:** Fatma Kilinc, Matthias Setzer, Bedjan Behmanesh, Daniel Jussen, Florian Gessler, Fee Keil, Volkhard A. J. Kempf, Johanna Kessel, Marcus Czabanka, Vincent Prinz

**Affiliations:** 1 Department of Neurosurgery, Goethe University Hospital, Schleusenweg 2-16, 60528 Frankfurt Am Main, Germany; 2 Department of Neurosurgery, University Medicine of Rostock, Rostock, Germany; 3 Department of Diagnostic and Interventional Radiology, Goethe University Hospital Frankfurt, Frankfurt Am Main, Germany; 4 Department of Medical Microbiology, Goethe University Hospital, Frankfurt Am Main, Germany; 5 Department of Medicine, Infectious Diseases Unit, Goethe University Hospital, Frankfurt Am Main, Germany

**Keywords:** Health care economics, Health services, Quality of life

## Abstract

Outpatient parenteral antimicrobial therapy (OPAT) is a cost-effective method of administering intravenous antimicrobial therapy. Although OPAT is well established in the UK and US healthcare systems, few centres in Europe perform it. Here we analysed OPAT for the treatment of patients with spinal infections at our institution. In this retrospective study, patients with spinal infection who required intravenous (i.v.) antimicrobial treatment between 2018 and 2021 were analysed. The duration of short-term antimicrobial treatment for skin and soft tissue infections and complex infections requiring long-term antimicrobial treatment, such as spinal bone or joint infections, were analysed. All patients were discharged with a peripherally inserted central catheter (PICC) line. Prior to discharge, all patients received training in the safe administration of their medications via the PICC line. The duration of OPAT and the rate of readmission after OPAT were analysed. For this study a total of 52 patients who were treated via OPAT due to spinal infections were analyzed. In 35 cases (69.2%) complex spinal infection was reason for i.v. antimicrobial therapy. Surgery was required in 23 of these 35 patients (65.7%). The average hospital stay for these patients was 12 ± 6 days. The remaining 17 patients were treated for an infection of the soft tissue or the skin and hospital stay for these patients was on average 8 ± 4 days. Gram-positive organisms were isolated in 64.4%. *Staphylococcus aureus* followed by other *Staphylococcus species*, was the most common detected organism. After discharging i.v. antimicrobial treatment was given for an average of 20 ± 14 days. The duration of antimicrobial treatment for soft tissue was 10.8 ± 8 days, and for complex infections 25.1 ± 18 days. The mean follow-up was 21 ± 14 months. There was one case of readmission due to treatment failure. There were no difficulties encountered in implementing OPAT. OPAT is a feasible and effective option for delivering intravenous antimicrobial therapy to patients with spinal infections who can be managed without hospitalisation. OPAT offers patient-centred treatment at home while avoiding the risks associated with hospitalisation, with high levels of patient satisfaction.

## Introduction

Outpatient parenteral antibiotic therapy (OPAT) is a rapidly expanding and evolving treatment option that allows patients to receive parenteral antimicrobials at home for many infectious diseases. A number of studies have shown that OPAT is a safe and effective therapy programme that allows patients to complete their treatment safely and effectively in the comfort of their own home^1–5^. In addition, it offers a number of benefits to both the patient as well as the health care provider through reduced length of stay in hospital, improved patient satisfaction and cost reduction^3–5^. Although OPAT is well established in the United Kingdome and US healthcare systems, so far there are only a few centers performing it in Germany/Europe and limited information on its prevalence^5–9^.

The efficacy and safety of OPAT in the treatment of infections such as endocarditis or pneumonia has been demonstrated in previous studies^10–12^. Although the incidence of spinal infections such as spondylodiscitis has increased in Western countries over the past few decades, and OPAT clearly has the potential to reduce the length of hospital admissions, few studies have investigated its safety and efficacy^12–15^.

Antibiotic regimens for complex spinal infections, such as spondylodiscitis or spinal empyema, require a total parenteral and oral treatment duration of 8–12 weeks^16–18^. Although OPAT is a highly attractive option for reducing the length of hospital stay, the number of hospital-related complications and the clinical benefits of avoiding hospitalisation as well as keeping care closer to home, so far there are only a few centers perform it.

## Methods

This retrospective study analysed patients with spinal infections who required i.v. antimicrobial treatment via OPAT between 2018 and 2021 and was performed at the neurosurgical department of Goethe-University Frankfurt. All patients were eligible for outpatient i.v. antibiotic therapy. Patients with spinal infections requiring only oral therapy were not included in the study.

Patient data were collected from the electronic patient records. The analyzed variables included demographics like age and sex, charlson comorbidity index (CCI), infectious disease diagnosis and treatment characteristics especially antimicrobial name, class and duration. Outcome like readmission and patients satisfaction were analyzed as well. Antibiotic-resistant organisms were defined as methicillin-resistant *Staphylococcus aureus* (MRSA), vancomycin-resistant enterococcus (VRE) and extended-spectrum B-lactamase Enterobacterales.

All diagnosed spinal infections were defined into two infection categories such as infections requiring short-term antimicrobial treatment (up to 7–10 days) such as skin and soft-tissue infections and long-term antimicrobial treatment (over 10 days) such as bone and joint infections. If bacteria were identified, empirical broad spectrum antibiotic therapy was changed to targeted culture-specific sensitive antibiotic therapy. Consultation with an infectious disease team was performed prior to antimicrobial therapy. Reports of laboratory values and changes in orders were made by phone, email or in person.

For all included patients, self-administered OPAT (S-OPAT) was applied in which the healthcare professional assists the patient and/or family members with the antibiotics schedule without the necessity for the healthcare personnel to be physically present at the home during the antibiotic administration. Patients were enrolled in the OPAT service after medical assessment if an infection requiring intravenous antibiotics were diagnosed, either for a short time or with a predetermined treatment duration. All included patients were medically stable and capable to understanding and consenting OPAT with safe social circumstances. Before discharging all patients received a peripherally inserted central catheter (PICC) line and in addition a training session for safely administering their medication via the PICC-line.

In order not to miss complications patients attended a post-discharge clinic at least once a week for clinical and laboratory checks^19^. This included weekly dressing changes and line care. Patients were trained to apply antibiotics to their PICC line at home, start the infusion process and disconnect it from the PICC line.

To assess patient satisfaction after OPAT standardised interviews were conducted during follow up using a structured, one-time, detailed questionnaire asking patients about any problems during i.v.-antimicrobial therapy.

### Ethical statement

This study was approved by the local ethics committee of the Goethe University Frankfurt am Main, the methods were carried out in accordance with the relevant guidelines and regulations. Informed consent was obtained in each case.

## Results

We identified a total of 52 patients with a median age of 52 years (range 27–85 years), who were treated via S-OPAT due to spinal infection between 2018 and 2021.

The gender distribution of the cohort was predominantly male, with 63.5% male (mean age 52). All diagnosed spinal infections were defined into two infection categories, such as infections requiring short-term antimicrobial treatment (up to 7–10 days) such as skin and soft-tissue infections, and long-term antimicrobial treatment (over 10 days), such as bone and joint infections. Their demographics and clinical characteristics are shown in Table 1.

**Table 1:** Characteristics of outpatient parenteral antibiotic therapy for patients with long-term treatment.

Sex	n = 35 (%) long-term treatment
Female	9 (25.7)
Male	26 (74.3)
Mean age, yrs	53
Charlson Comorbidity Index (CCI)	
0	1 (2.9)
1	4 (11.4)
2	10 (28.6)
3	13 (37.1)
4	5 (14.3)
5	2 (5.7)
Laboratory findind on admission	
CRP	11.87 ± 4.41(mg/dl)
WBC	5.6 ± 1.18(/μl)
Laboratory findind during last follow up	
CRP	1.45 ± 0.41(mg/dl)
WBC	5.6 ± 1.18(/μl)
OPAT antibiotics (number of episodes (%))	
Cefazolin	15 (42.9)
Fosfomycin	6 (17.1)
Meropenem	2 (5.7)
Vancomycin	7 (20)
Penicillin	2 (5.7)
Ceftriaxone	7 (20)
Daptomycin	2 (5.7)
Gentamycin	1 (2.9)
Ciprofloxacin	3 (8.6)
Clindamycin	4 (11.4)
Duration of treatment (mean, days)	
Surgical treated patients	
Intra-hospital	9 ± 5.1
OPAT	25.1 ± 18
Conservative treated patients	
Intra-hospital	9 ± 5
OPAT	25.1 ± 18
Conservative	
Intra-hospital	9 ± 5
OPAT	14 ± 9.4

Complex spinal infections such as spondylodiscitis or spinal empyema were found in 35 cases (69.2%). In five cases (14.3%), the infection occurred after surgery, while the remaining 30 patients had no previous surgery. In 23 of these 35 patients (65.7%), treatment consisted of surgical debridement of the infected vertebrae and disc material, fusion and spinal instrumentation. The average length of hospital stay after surgical treatment for this cohort was 12 ± 6.3 days. In the remaining patients (12 patients) surgery was not necessary and conservative therapy with i.v.-antibiotic-therapy alone was sufficient. The average hospital stay for this cohort was 9 ± 5 days.

The remaining patients (17 patients) were treated for soft tissue or skin infection (Table 2). All of these patients had previously undergone surgery. The average hospital stay for this cohort was 8 ± 4 days.

**Table 2:** Characteristics of outpatient parenteral antibiotic therapy for patients with short-term antimicrobial treatment.

Sex	n = 17 (%)short-term treatment
Female	10 (58.8.7 )
Male	7 (41.2)
Mean age, yrs	50
Charlson Comorbidity Index (CCI)	
0	1 (5.9)
1	2 (11.8)
2	2 (11.8)
3	8 (47.1)
4	3 (17.6)
5	1 (5.9)
Laboratory findind on admission	
CRP	5.32 ± 2.11(mg/dl)
WBC	8 ± 2.29(/μl)
Laboratory findind during last follow up	
CRP	1.15 ± 0.29(mg/dl)
WBC	5.0 ± 1.09(/μl)
OPAT antibiotics (number of episodes (%))	
Cefazolin	7 (41.2)
Fosfomycin	6 (35.3)
Meropenem	4 (23.5)
Vancomycin	1 (5.9)
Penicillin	1 (5.9)
Ceftriaxone	1 (5.9)
Ertapenem	2 (11.8)
Gentamycin	1 (2.9)
Clindamycin	1 (5.9)
Duration of treatment (mean, days)	
Surgical treated patients	
Intra-hospital	8 ± 4
OPAT	10.8 ± 8

The majority of patients (54.3%) with long-term antimicrobial treatment were treated with two or more drugs for the duration of OPAT. The most commonly prescribed antimicrobials was cefazolin (41.2%), followed by ceftriaxone (20%) and vancomycin (20%).

The majority of patients with short-term antimicrobial treatment were treated with one drug for the duration of OPAT. The most commonly prescribed antimicrobial was cefazolin (41.2%) followed by fosfomycin (35.3%).

Gram-positive organisms were isolated in most cases (64.4%). The most common pathogens were *Staphylococcus aureus,* followed by other *Staphylococcus species*. In two cases, 3 multi-resistant gram-negative pathogenes (MRGN) pathogens were detected. After identification of the microorganisms, a bactericidal and resistogram antibiotic was administered (Table 3).

**Table 3:** Aetiological pathogens isolated from blood cultures or intraoperative specimens and outcome after OPAT.

Organism isolated	n(%)
*Staphylococcus aureus*	29 (55.8)
Methicillin-sensitive *S aureus*	27 (51.9)
Methicillin-resistant *S aureus*	2 (3.8)
Other *Staphylococcus species*	8 (15.4)
*Streptococci*	7 (13.5)
Culture negative	8 (15.4)

For all included patients, the average duration of OPAT after discharge was 41.3 ± 16.4 days. The duration for short-term antimicrobial treatment was 10.8 ± 8 days, and for complex infections 25.1 ± 18 days. One patient had to be readmitted due to treatment failure. All patients had regular follow-up and laboratory monitoring after discharge. No PICC line complications were detected. No adverse effects or complications regarding laboratory parameters were observed.

To assess patient satisfaction, standardised interview with a questionnaire were conducted at the last clinical follow-up after completion of i.v. antibiotic therapy. 98.1% of patients reported that they were satisfied with i.v. therapy at home and 82,7% had no problems with managing the antibiotic therapy at home. In 67.3% patients reported that they were able to carry out tasks at home on their own during treatment. 30.8% of patients were able to return to work and 92.3% reported having good health care. Only in one case was the patient dissatisfied because he had to be readmitted and undergo a second operation. (Table 4).

**Table 4:** Outcome.

	n(%)
Patients satisfaction during OPAT	51 (98.1)
Readmission during OPAT	1 (1.9)
Managing to do’s alone at home	35 (67.3)
Return to work during OPAT	16 (30.8)
Good health care	48 (92.3)
Managing antibiotic therapy at home	43 (82.7)

## Discussion

The main findings of this study are OPAT is a effective option for delivering intravenous antimicrobial therapy for patients with spinal infections who can be managed without hospitalisation. It allows patient-centered care to be provided at home, avoiding the risks associated with hospitalisation and improving patient satisfaction.

The aim of the present study was to evaluate the effect of outpatient parenteral antimicrobial therapy in patients with a spinal infection. Parenteral antibiotic administration for spinal infections usually requires inpatient treatment, also known as inpatient parenteral antibiotic therapy. In contrast to the models from the USA and the United Kingdom, and despite the described clinical benefits of avoiding hospitalisation and keeping care closer to home, there is still no uniformly established system of outpatient therapy centres for the parenteral administration of antibiotics OPAT in Germany^2,4,5,9^.

The incidence of spinal infections such as spondylodiscitis or spinal empyema has been increasing in Western countries over the past few decades^16,20,21^. The main reasons for this are demographic changes, increased life expectancy and improved imaging and clinical diagnostics. Furthermore, it may also be related to the increasing number of spinal surgeries and instrumentation. The median age of patients with spinal infections is increasing and a high number of these patients have significant comorbidities^22,23^. The desire to discharge patients with spinal infections from hospital at the earliest opportunity is further augmented by concerns over hospital-acquired infection, especially in those receiving long courses of antimicrobials.

In Germany, most patients with spinal infections are treated according to guidelines with intravenous antibiotics followed by oral therapy. In hospitals where OPAT is not available, patients are treated as inpatients until i.v. therapy is completed.

Li et al. described 2019 in the OVIVA study that oral antibiotic therapy was not inferior to intravenous antibiotic therapy in terms of treatment failure at one year when used in the first 6 weeks of treatment for bone and joint infections^24^. All patients included in our study were treated with OPAT as indicated by clinical findings. To avoid treatment failure, antibiotic therapy was discussed with infectious disease colleagues and intravenous therapy was decided on the basis of the pathogen spectrum and the antibiogram. Patients treated orally only were not included in the study. Obviously, insurance coverage is an important factor, as the cost of OPAT is covered by insurance. If patients without insurance coverage require outpatient i.v.-therapy, the social security office ensured that the costs were covered before discharge.

The analysed results of this study show that, despite the gravity and complexity of spinal infection, it is possible to carry out the OPAT schedule successfully and efficaciously. This study also presents some of the first published data on the use of OPAT for spinal infection in Germany.

OPAT is primarily about delivering high quality patient centered care closer to home while avoiding inherent risks associated with hospitalisation. These positive healthcare findings should be utilized by OPAT clinician/healthcare managers and policymakers alongside the already powerful clinical effectiveness and patient safety data to drive further OPAT development in Germany.

Our data show that the OPAT service model used at our centre is clinically effective, with low rates of complications/readmissions and patient satisfaction.

In conclusion, the results of this study suggest that OPAT is a feasibility alternative to inpatient care for a wide range of infections in patients with spinal infections.

## Limitations

In addition to the sample size, other limitations of this study include the retrospective study design. Most patients in our study had a long follow-up. Our cohort may be subject to recall bias due to the time elapsed between our assessment and the time of OPAT. Possible complications or side effects of antibiotic therapy were not analysed. The only safety/efficacy outcome analysed was readmission. This is a significant limitation and should be noted.

## Supplementary Information

Supplementary Information.

## Data Availability

The datasets generated during and/or analyzed during the current study are available from the corresponding author on reasonable request.
