# Supplementary material for: Safety and feasibility of outpatient parenteral antimicrobial therapy for patients with spinal infection
Source: Sci Rep. 2023 Apr 26;13:6863. doi: 10.1038/s41598-023-33502-7 (PMC10133347; doi:10.1038/s41598-023-33502-7)
Supplement: Supplementary file 1 — Supplementary Information. [file 41598_2023_33502_MOESM1_ESM.docx]

**Questionnaire**

**Answering the following questions is voluntary and serves to record patient satisfaction.**

**All information will be treated confidentially.**

**Mark only one answer option per area.**

1. mark your current health status


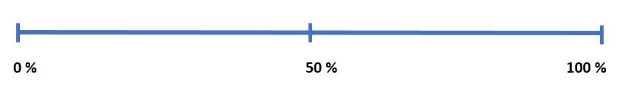


100% means: best state of health

0% means: worst state of health

1. mark the corresponding number

Pain intensity


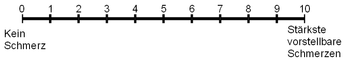


No pain worst pain

Please answer with „yes“ or „no”:

- Were you satisfied with the outpatient i.v. therapy?
- Can you manage your household?
- If you are employed, have you started working again?
- Do you have any problems with managing the antibiotic therapy?
- Do you feel you have good health care?
